# A Comparison of In Vitro Points of Departure with Human Blood Levels for Per- and Polyfluoroalkyl Substances (PFAS)

**DOI:** 10.3390/toxics12040271

**Published:** 2024-04-05

**Authors:** Richard S. Judson, Doris Smith, Michael DeVito, John F. Wambaugh, Barbara A. Wetmore, Katie Paul Friedman, Grace Patlewicz, Russell S. Thomas, Risa R. Sayre, Jennifer H. Olker, Sigmund Degitz, Stephanie Padilla, Joshua A. Harrill, Timothy Shafer, Kelly E. Carstens

**Affiliations:** US Environmental Protection Agency, Research Triangle Park, NC 27711, USA; smith.doris@epa.gov (D.S.); devito.michael@epa.gov (M.D.); wambaugh.john@epa.gov (J.F.W.); wetmore.barbara@epa.gov (B.A.W.); paul-friedman.katie@epa.gov (K.P.F.); patlewicz.grace@epa.gov (G.P.); thomas.russell@epa.gov (R.S.T.); sayre.risa@epa.gov (R.R.S.); olker.jennifer@epa.gov (J.H.O.); degitz.sigmund@epa.gov (S.D.); padilla.stephanie@epa.gov (S.P.); harrill.joshua@epa.gov (J.A.H.); shafer.tim@epa.gov (T.S.); carstens.kelly@epa.gov (K.E.C.)

**Keywords:** PFAS, biomonitoring, in vitro, margin of exposure, chemical prioritization

## Abstract

Per- and polyfluoroalkyl substances (PFAS) are widely used, and their fluorinated state contributes to unique uses and stability but also long half-lives in the environment and humans. PFAS have been shown to be toxic, leading to immunosuppression, cancer, and other adverse health outcomes. Only a small fraction of the PFAS in commerce have been evaluated for toxicity using in vivo tests, which leads to a need to prioritize which compounds to examine further. Here, we demonstrate a prioritization approach that combines human biomonitoring data (blood concentrations) with bioactivity data (concentrations at which bioactivity is observed in vitro) for 31 PFAS. The in vitro data are taken from a battery of cell-based assays, mostly run on human cells. The result is a Bioactive Concentration to Blood Concentration Ratio (BCBCR), similar to a margin of exposure (MoE). Chemicals with low BCBCR values could then be prioritized for further risk assessment. Using this method, two of the PFAS, PFOA (Perfluorooctanoic Acid) and PFOS (Perfluorooctane Sulfonic Acid), have BCBCR values < 1 for some populations. An additional 9 PFAS have BCBCR values < 100 for some populations. This study shows a promising approach to screening level risk assessments of compounds such as PFAS that are long-lived in humans and other species.

## 1. Introduction

Per- and polyfluoroalkyl substances (PFAS) are a widely used class of chemicals with unique properties due to their fluorinated state [1,2]. Uses include non-stick coatings, stain-resistant finishes for fabrics, firefighting foams, paint components, toilet paper coatings, and others. PFAS are a structurally diverse class of chemicals, with some substances showing stability in the environment and some showing bioaccumulative properties (or potential), with half-lives in humans up to several years [3,4]. There is increasing evidence that some PFAS cause health effects, including immunotoxicity, developmental and reproductive effects, increased BMI, decreased birthweight, and cancer [2,5,6,7,8,9,10,11,12,13,14,15,16,17,18]. These conclusions arise from epidemiology studies that have shown correlations between the levels of PFAS in the environment (e.g., drinking water) and levels of incidence of certain health effects (many of the previously cited references), plus experimental studies on animals [19].

Thousands of unique PFAS have been produced for use in consumer goods or exist as byproducts of chemical manufacturing or degradation in the environment [20,21] (see lists on the EPA CompTox Chemicals Dashboard, e.g., reference [22]), but little is known regarding the extent to which humans and environmental species are exposed. At the same time, only a small fraction of PFAS have been tested for theirpotential hazards in experimental animals [23]. Without this kind of information, making informed risk assessment decisions is difficult. To help characterize the landscape of PFAS hazards, we carried out a study in which ~150 PFAS of diverse structural classes were tested in eight sets of in vitro bioactivity assays, including whole genome transcriptomics, a zebrafish embryo developmental toxicity assay, and a developmental neurotoxicity battery using primary rat and human inducible pluripotent (iPS)-derived neural cells. One result of this work is a set of in vitro points of departure (PODs) for each of the tested chemicals. These in vitro PODs provide the concentration at which biological effects occur and can help estimate a lower bound on the concentration at which a chemical could potentially cause adverse effects in a whole animal or human.

Because of concerns about the risks posed by PFAS, and the tendency for some of them to bioaccumulate, a large number of biomonitoring studies have been carried out to measure the concentrations of multiple PFAS in humans and other species in several matrices (e.g., blood, urine, breast milk, semen, tissues in fish, and other aquatic organisms). These data streams (human blood concentrations and in vitro PODs) can be combined to estimate what we call the Bioactive Concentration to Blood Concentration Ratio (BCBCR), for chemicals with both data sets available. The BCBCR is similar to a margin of exposure (MoE). Ludwicki et al. [24] used a similar approach to calculate a Hazard Quotient (HQ, [25]) for PFOA (Perfluorooctanoic Acid) and PFOS (Perfluorooctane Sulfonic Acid) in several European populations by comparing measured levels in blood to blood-level PODs from cynomolgus monkeys. Typically, the HQ instead compares doses (e.g., known hazardous doses vs. exposure doses). Another approach to using biomonitoring data in risk assessment is the biomonitoring equivalent approach, which compares (human) internal concentrations to concentrations that cause effects (e.g., the blood concentration at an animal-derived lowest observed adverse effect level (LOAEL)) [26,27,28]. Note that the compound seen in blood or other tissues may not be the chemical to which the organism was initially exposed but instead may be an environmental or metabolic breakdown product.

The method described here is most appropriate for a class of chemicals like many PFAS, which, due to their slow clearance, can be detected at relatively constant blood levels across multiple independent blood biomonitoring studies. Low turnover makes interpretation of biomonitoring data simpler for these PFAS in contrast to nonpersistent chemicals, which require robust biomonitoring programs to evaluate biomarkers of exposure and effect because of greater longitudinal variability [29]. In this study, we have calculated BCBCR values for 31 chemicals with in vitro bioactivity data, human biomonitoring data, and in vitro-derived predictions of blood-to-plasma concentration ratios. The in vitro data are taken from a battery of cell-based assays, mostly run on human cells. The key result from each assay is the concentration in cells that will cause a biological perturbation. In general, there is no direct link between the in vitro bioactivity detected by an assay and a specific apical in vivo toxicological effect. However, previous studies have demonstrated that in vitro bioactivity provides a conservative estimate of the dose-causing toxicological responses in traditional animal-based studies [30]. This work demonstrates the overall approach and provides one approach for carrying out screening-level risk assessments for other PFAS.

The aims of this study are to (1) describe and illustrate the BCBCR method; (2) apply the method to all PFAS for which data are available; (3) provide a ranking of these PFAS in terms of this risk-based metric; and (4) enumerate sources of uncertainty in the BCBCR values.

## 2. Materials and Methods

### 2.1. Biomonitoring Data

Human biomonitoring data were collected from 247 published studies. These are documented in Supplemental Material S1 in two forms. The first is a text document with references, and the second is an Excel file with URLs and additional columns of information regarding the data set. One previously unpublished set of biomonitoring data comes from the 3M Company in the form of a collection of documents provided to the US EPA under a consent order. These documents are provided as part of Supplemental Material S1. Each data set is characterized by the sampling location (country, state, region, or city) and a brief statement about the cohort, especially whether they were suspected of being exposed to PFAS compounds (e.g., factory workers) or were a general population. Note that a single document may yield more than one data set, for instance, one for children and one for adults. Data were extracted from the original study reports into the ACToR (Aggregated Computational Toxicology Resource) [31] database (now included in the CompTox Chemicals Dashboard [32]), and then reexported in a standardized format. Chemicals were mapped by name or Chemical Abstracts Registry Number (CASRN) to substances in the DSSTox database [33] and assigned DSSTox Substance IDs (DTXSID). Concentrations in several matrices were available in these studies (whole blood, serum, plasma from both adults, and cord blood). All concentrations were converted to ng/mL. Each study reported one or more concentration metrics for the population tested. The metrics are the 5th percentile, 10th percentile, 25th percentile, 50th percentile, 75th percentile, 90th percentile, 95th percentile, 98th percentile, 99th percentile, maximum, mean, median, and minimum. Studies reported various types of means (mean, arithmetic mean, geometric mean, and average), and all of these are designated here as “means”. In addition, most studies reported a limit of detection (LOD) and/or a limit of quantitation (LOQ). Data for all available metrics were included in the analyses. The matrix and metric values (see below) were manually extracted from the documents or notes in the ACToR database. Each ACToR data set is labeled by a code, the source_name_aid, or SNAID. This code is used to link the details of the data sets to the individual data points. There are a total of 294 data sets and 38,662 individual values from different chemicals, sources, metrics, subpopulations, and matrices. Because these data were processed through multiple steps, both computational and manual, a QC check was performed by checking the final values against the source document for all values > 100 ng/mL (all but one in vitro POD were above this level). All values from 71 data sets were correct, while some values from six data sets had the data type incorrectly mapped and were actually the number of study participants or years of data collection. There are a small number of duplicates in the data set because some studies are already summaries, and, for instance, multiple data sets report selected NHANES data. No attempt was made to remove duplicate records from the current data set. Details of the data transformations are included in Supplemental Material S2. All of these processes are encoded in an R language package (see Supplemental Material S3).

### 2.2. In Vitro Toxicokinetic (TK) Data and Partition Modeling

Biomonitoring studies measured concentrations in plasma, serum, or whole blood matrices, but for consistency, values are converted to plasma concentrations, which is the matrix used for performing toxicokinetic calculations. To convert values in matrices other than plasma, we used partition coefficients [34] taken from the open-source R software package, httk (version 2.3.0). The steady-state blood-to-plasma concentration ratio is predicted with a calibrated version of the Schmitt 2008 algorithm using the function *httk::get_rblood2plasma()* [34,35]. Chemical-specific in vitro plasma protein binding was recently measured and reported for ~120 PFAS compounds [36,37,38]. *Httk* includes in vivo measured blood:plasma ratios for four PFAS; for these chemicals, the in vivo values were used in place of the in vitro-derived predictions [39]. We assume that c(plasma) = c(serum). For whole blood, c(plasma) = c(whole blood)/blood-to-plasma ratio, as provided by the *httk* package using a blood-to-plasma partition coefficient. For chemicals with all data except the blood-to-plasma partition coefficient, this value is set to 0.5. Of the 25 chemicals with calculated or measured partition coefficients, 22 were between 0.5 and 0.6. We assume that the measured c(plasma) is the total concentration and not just the free concentration. Some studies reported values from cord blood, cord serum, cord plasma, or blood spots, and these were treated, respectively, as whole blood, serum, plasma, and whole blood. Partition coefficients for humans, and rats are given in Supplemental Material S4.

### 2.3. In Vitro Bioactivity Data

The in vitro bioactivity data are derived from a set of ~150 PFAS compounds that were processed through eight sets of assays. The number of PFAS screened differed slightly by technology due to concurrent analytical quality control testing. All of the PFAS reported here passed analytical QC [36], which indicates that the samples tested had the intended chemical identity. The in vitro assays are described briefly here, and references provide more detail. The assays are grouped into “assay sets”, where a set contains all assays from a single vendor or source with distinct assay technology and/or bioactivity type and cell type. For each chemical, there is a POD for each assay set. Unless otherwise noted, the set-level POD is the lower 5th percentile of the distribution of all PODs for that chemical and assay set for active assays. The minimum POD for the chemical is the minimum of the set-level PODs. If all assay endpoints for a technology are inactive, the returned set-level POD is set to 1000 μM. For clarity, the assay set-level PODs are indicated by POD_set_ and the chemical-level PODs by POD_chemical_. Except where noted below, the maximum tested concentration was 100 μM. The in vitro PODs are given in Supplemental Material S5.

#### 2.3.1. ACEA: (ACEA Biosciences, San Diego, CA; [40,41])

This assay is a functional screen for estrogen-mimicking substances, and uses a real-time impedance measurement over a 72 hr exposure period during which impedance increases in response to increases in estrogen receptor-dependent cell proliferation in the human breast carcinoma cell line, T-47D. There are two assay endpoints: one for estrogen receptor-dependent cell proliferation and one for decreased cell viability in the system. The ACEA POD is equal to the minimum ToxCast Pipeline (tcpl, version 2.1.0) [42] 50% activity concentration (AC50) for these two endpoints with an active hitcall. For ACEA, the maximum tested concentration was 300 μM, pending solubility limitations.

#### 2.3.2. ATG: (Attagene, Morrisville, NC; [41,43])

This platform measures a large number of ligand-activated nuclear receptor and other transcription factor activities representing diverse physiological processes including metabolism and fatty acid regulation, endocrine activity, oxidative stress, and lipid peroxidation using two assay modes (cis and trans) in the H19 subclone of HepG2 cells reflecting elevated cytochrome P450 expression. There are 81 individual targets in this multiplexed panel. The top target assay concentration was 300 µM, pending solubility limitations.

#### 2.3.3. BSK: (BioSeek, Now BioMAP, Diversity plus Panel, [44,45,46,47])

This assay set consists of 12 human primary cell systems that model potential tissue and disease responses, including vascular, immune, skin, lung, and general tissue responses, via stimulation of the mono- or co-culture systems to pathophysiologically relevant states. There are a total of 148 individual assay components that report hitcall and lowest effect level (LEL), which is the lowest discrete concentration at which a significant change in response from baseline is seen. A separate POD is derived for each of the 12 assay sets constituting different primary cells or co-cultures.

#### 2.3.4. DNT

This assay battery was designed to detect chemicals with potential for developmental neurotoxicity (DNT; see Carstens et al. [48] for detailed experimental design and tcpl pipeline methods). The DNT assay battery included four assays from two technologies: microelectrode arrays (MEA) [49] and high-content imaging (HCI) [50]. The MEA network formation assay (NFA) [49,51] measured changes in neuronal electrical activity in rat primary cortical neurons over a 12-day exposure period. The NFA included 17 parameters measuring decreased neuronal activity and two cytotoxicity endpoints. The HCI technology included three assays: one measuring neurite outgrowth (NOG) in human ‘iCell Gluta’ neurons, one measuring proliferation in human neural progenitor cells (hNP1), and one measuring apoptosis in the hNP1 cells. The HCI assays ranged from 1 to 2 days of exposure, and each included a measure of cytotoxicity. Chemical concentration response data were normalized and curve-fitted using tcpl to identify active or inactive chemicals. Several criteria were used to filter low-confidence concentration response curves: (1) curves with ≥3 caution flags, (2) positive curves with a model top less than or equal to 1.2 times the cutoff and a resultant AC50 less than the concentration range screened; and (3) any hitcalls of −1, indicating the concentration series had fewer than four concentrations. A hitcall was set to zero, and AC50 values were set to ‘NA’ if any if these three criteria were met.

#### 2.3.5. HTPP: (High-Throughput Phenotypic Profiling with the Cell Painting Assay [52,53,54])

This high-content imaging assay measures phenotypic changes in cell morphology in cells labeled with fluorescent markers for a variety of organelles (nucleus, nucleoli, endoplasmic reticulum, Golgi complex, plasma membrane, cytoskeleton, and mitochondria). The assay was run in MCF7 (breast adenocarcinoma) and U-2 OS (osteosarcoma) cell lines. Additionally, HTPP includes a cell viability endpoint. The outputs include a cell viability BMC (benchmark concentration), 1300 individual feature-level BMCs (benchmark concentration), 49 category-level BMCs, and one global BMC as described [53]. BMCs and hitcalls are derived using the *tcplfit2* method [55]. The POD used here is the lowest of the category BMCs for each cell type.

#### 2.3.6. HTTr: (High-Throughput Transcriptomics with the TempO-Seq Human Whole Transcriptome Assay [56,57])

This assay measures gene expression changes using whole transcriptome targeted RNA-Seq in HepaRG (liver) and U-2 OS cell lines. Raw data are converted to log2 fold change values for each gene, and then these are aggregated into changes in gene sets or signatures as described [57]. A BMC is derived for each chemical-signature pair. The PODs used here are the lower 5th percentile of the BMCs for active signatures in each cell type.

#### 2.3.7. Thyroid

In order to rapidly evaluate the potential impacts of PFAS on the thyroid axis, we employed medium-throughput assays that use recombinase enzymes [58]. The assays reported here test seven Molecular Initiating Events (MIEs) in the thyroid Adverse Outcome Pathways (AOPs) network [59]. This suite of assays covers critical pathways within the thyroid axis, including deiodinase enzymes (Human Deiodinase 1,2, and 3 [DIO], Human Iodotyrosine deiodinase [IYD] [60,61], human thyroid peroxidase [TPO] [62], and thyroid hormone plasma-binding proteins transthyretin [TTR], and thyroxine-binding globulin [TBG] [63]. These seven MIEs link to 16 known or putative pathways in the AOP wiki [64]. For the DIO, IYD, and TPO assays, the maximum concentration was 300 μM in single-point and 200 μM in multi-concentration runs. For the TBG and TTR assays, the single and multi-point concentration maximum concentrations were both 150 μM. (DIO, IYD, and TPO used recombinant enzymes produced in-house; TGB, and TTR used purified human enzymes (purchased).

#### 2.3.8. Zebrafish

This is a zebrafish embryotoxicity assay that measures lethality and malformations (hatching status, swim bladder inflation, edema, abnormal spinal or craniofacial structure, blood pooling, or changes in pigmentation) in concentration-response format. Each endpoint is assigned a benchmark concentration (BMC), and the POD is the lowest of the BMC values. Standard protocols have been followed [39,65]. Concentration-response modeling was carried out using the R package *tcplfit2* [55]. Full details of the assay are available in Britton et al. [in preparation].

### 2.4. BCBCR Calculation

The BCBCR is the ratio of the in vitro POD_chemical_ (converted to ng/mL) divided by the plasma concentration, also in ng/mL. Values < 1 occur when the plasma concentration exceeds the in vitro POD_chemical_, indicating that bioactivity could occur at that plasma concentration. The biomonitoring data provide the total concentration in a sample and not just the free (plasma-unbound) concentration. The in vitro PODs are derived based on the nominal (total) concentrations delivered to the testing well at which bioactivity is observed. To evaluate target tissue exposures and effects, one would ideally convert a plasma concentration to a tissue concentration using a plasma-tissue partitioning model similar to what has been described for other drugs and non-drugs [28,29]. Although attempts were made for PFAS, it was concluded that the resulting partitioning predictions were highly uncertain when evaluated using available empirical data, likely due to the C:F backbone imparting unique partitioning that hindered the development of meaningful conversion factors for PFAS. Therefore, the nominal BCBCR approach was used. The potential impacts of this approach are addressed in more detail in the discussion.

### 2.5. In Vivo Data with Internal Concentrations in Rats

We include in vivo data derived from two NTP toxicology studies [6,7] in which a set of seven PFAS were tested in Sprague-Dawley rats. These were 28-day studies with oral gavage dosing using an equal number of male and female rats 10–11 weeks of age. The chemicals are PFOA (perfluorooctanoic acid), PFOS (Perfluorooctanesulfonic acid), PFDA (Perfluorodecanoic acid), PFBS (Perfluorobutanesulfonic acid), PFNA (Perfluorononanoic acid), PFHxA (Perfluorohexanoic acid) and PFHxSK (Perfluorohexane sulfonate potassium salt). Blood concentration data were available for PFOA, PFOS, PFDA, PFBS, PFNA, and PFHxA. It is also available in the acid form of PFHxSK. Plasma levels were measured at each testing concentration at the end of this study. Lowest effect levels (LELs) in mg/kg-day were determined for the following effects: liver weight, relative liver weight, kidney weight, relative kidney weight, decreased hematocrit, decreased cholesterol, decreased T3, decreased free T4, and decreased total T4. An LEL is the lowest dose at which there is a statistically significant difference in the parameter from that of control animals. For each chemical/sex combination, the lowest LEL was determined and assigned as the point of departure (POD_in vivo_). The plasma concentration (in ng/mL) at the lowest LEL was used in the remaining analyses. The complete in vivo data set is summarized in Table 1. These studies were selected because they use a single, standard protocol; they have blood levels measured as all chemical doses; and the chemicals overlap with our current study chemicals. A complete literature search for other such studies was not carried out.

## 3. Results

A total of 94 chemicals had biomonitoring data in at least one matrix, 31 of those had in vitro POD data, and this set of 31 also had blood-to-plasma partition coefficients. All 31 chemicals passed analytical QC [36]. Table 2 lists this set of chemicals with their names, CASRN, DTXSID, and abbreviations.

**Figure 1:**
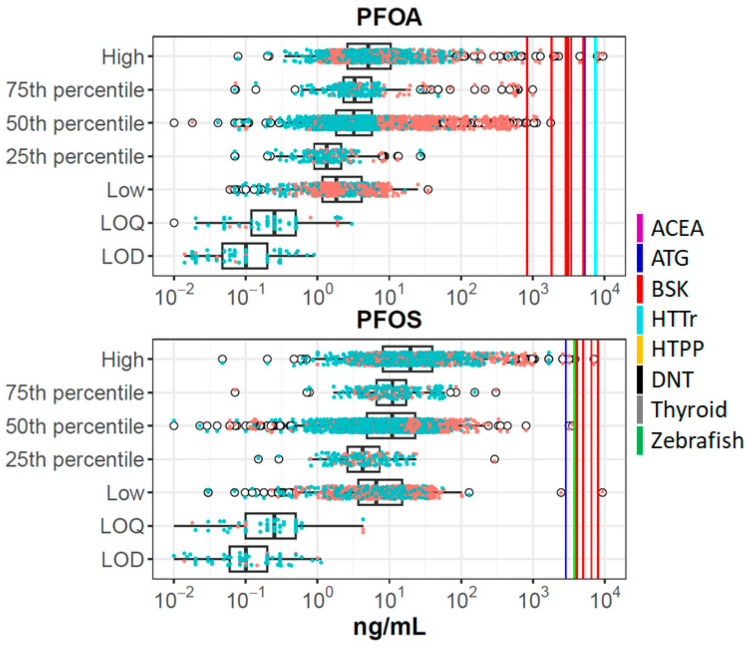
Summary of in vitro and biomonitoring data for PFOA and PFOS. The boxplots show distributions of plasma concentrations from all studies for each chemical. The box-and-whiskers plot indicates the interquartile range (IQR) and 1.5 times the IQR. The open circles are points outside 1.5 times IQR. Overlaid on this are data points for each individual population and metric, colored orange (exposed populations) or blue (general populations). The vertical solid lines show POD_set_ values as indicated in the legend (ACEA: violet; ATG: blue; BSK: red; HTTr: cyan; HTPP: orange; DNT: black; Thyroid: gray; Zebrafish: green). For these chemicals, some of the assay technologies (e.g., DNT) were inactive, so no corresponding line is shown. The metric groups on the y-axis are High (>75% percentile, including maximum); 75th percentile; 50th percentile (50th percentile, mean, median); 25th percentile; and Low (<25th percentile, including minimum). LOQ is limit of quantification and LOD is limit of detection.

For each of these chemicals, we combined data from all sources and summarized it in plots such as Figure 1, showing data for PFOA and PFOS. Corresponding figures for all chemicals are provided in Supplemental Material S6. Different studies report different metrics (e.g., one study will report a mean, while another will report different percentiles), and because some sources combine data from multiple experimental studies, there may be more than one mean, median, etc. The boxplots show raw data from the biomonitoring studies (concentrations in all matrices, converted to plasma concentrations). Here one can see the expected result that most values are above the LOD/LOQ, and that data from higher percentiles of distributions are above those from lower percentiles. Individual data points are indicated by the scattered points, where orange points are taken from exposed populations and blue points from general populations. As expected, the higher values are enriched by data from exposed populations. The in vitro set-level PODs (POD_set_) are indicated by the vertical lines, with different colors indicating different technologies, as defined in the figure legend. PFOA and PFOS are the only chemicals with any BCBCR values < 1, and in all cases, these data points are from exposed populations, typically for the median or greater within those exposed populations.

**Figure 2:**
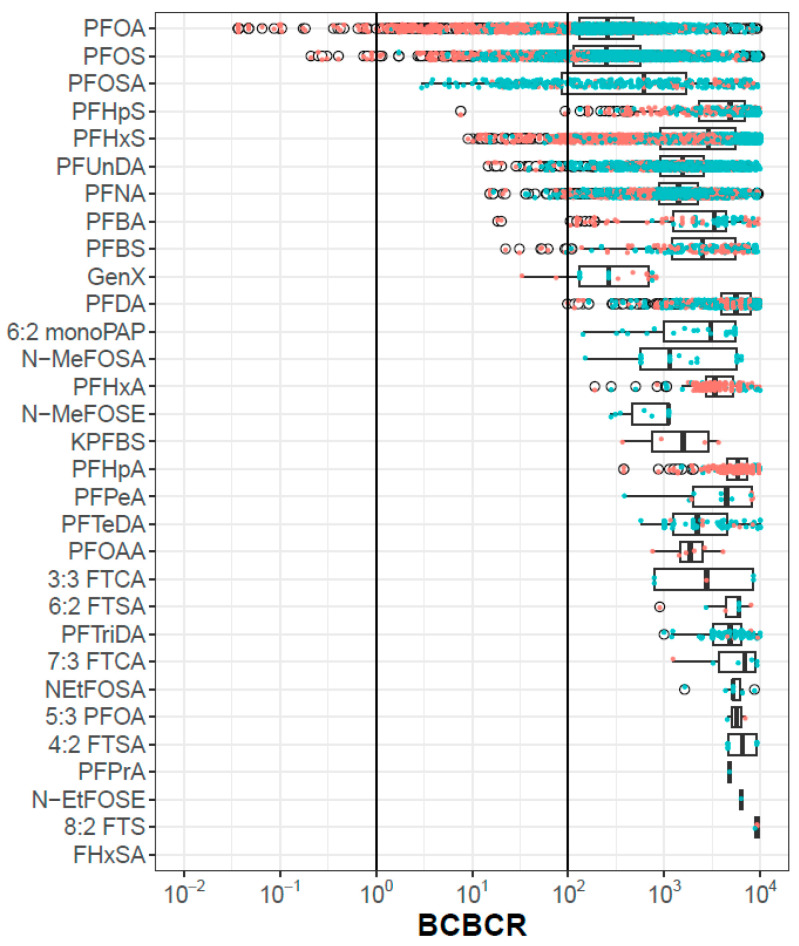
BCBCR values as a function of chemical, metric and population. Each point is one population-metric value for one chemical. Points colored orange are from exposed populations and those colored blue are from general populations. The box and whiskers indicate the inner quartiles and 1.5 times the IQR, respectively. The open circles are points outside 1.5 times IQR.

Figure 2 shows the BCBCR values for the 31 chemicals for all population metrics. Only PFOS and PFOA have any BCBCR values < 1, and PFOSA, PFHpS, PFHxS, PFUnDA, PFNA, PFBA, PFBS, GenX, and PFDA have at least one population/metric pair with a BCBCR <100. The discussion section will catalog various areas of uncertainty that could, in aggregate, reach a factor of 100. There is a trend that data from exposed populations (orange points) have lower BCBCRs than those from general populations (blue points), but this is not universally true, as exemplified by PFOSA. The bottom chemical in Figure 2 (FHxSA) has no BCBCR values < 10,000, and so no points are visible. The complete set of BCBCR values is provided in Supplemental Material S7. Among the BCBCR values < 100, the in vitro bioactivity assays driving the POD are ATG (PFOS, GenX, PFHxS), BSK (PFHpS, PFOA, PFBS, PFBA, PFNA, and PFUnDA), HTPP U2OS (PFDA), and zebrafish developmental toxicity (PFOSA).

The approach used in this analysis assumes that chemicals have long half-lives, so that blood concentrations are relatively stable over time. Only a few PFAS have measured half-lives, but a recent paper by Dawson et al. uses a QSAR model to predict human plasma half-lives for a large collection of PFAS [66]. The model does not provide a numeric half-life, but instead a class, which is one of <0.5 days, <1 week, <2 months, or >2 months. This model predicts that all but one of the 31 PFAS analyzed here have half-lives > 2 months [66]. The one exception is PFPeA, with a half-life of <1 week. This chemical has relatively low concentrations detected in any study, and (from Figure 2), there are no instances of a BCBCR < 1000. Chiu et al. recently published estimates of half-lives for PFOA, PFOS, PFNA, and PFHxS, all of which exceed several years [67].

**Figure 3:**
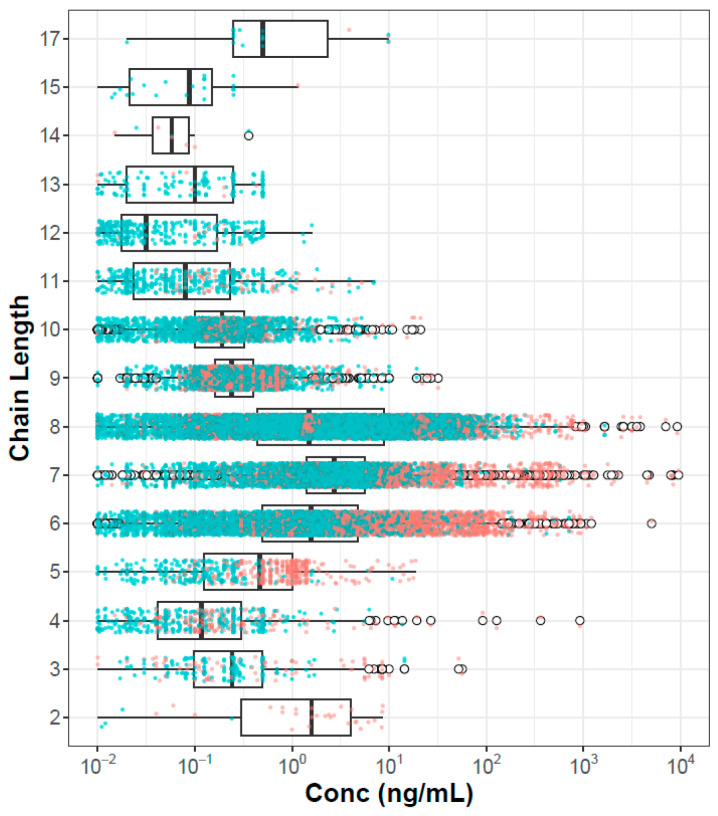
Plasma concentration values for all chemicals, populations, and metrics as a function of chain length. Points colored orange are from exposed populations, and those colored blue are from general populations. Box and whisker annotations are the same as in Figure 1 and Figure 2.

One hypothesis concerning PFAS risk is that hazard and potentially bioaccumulation are inherently driven by chain lengths. It may be that short-chain-length chemicals will be more easily cleared, and long-chain-length chemicals may be poorly bioavailable. This is consistent with findings in a recent evaluation of in vitro plasma protein binding of 67 PFAS, where lower binding was noted for PFAS with 11 or more carbons compared to those with 6–10 [36]. Figure 3 shows the plasma concentration data organized by chain length, where chain length is the maximum contiguous number of carbons that are fully fluorinated. Here we see that PFAS with intermediate chain length (6–8) clearly have higher concentrations than PFAS with shorter or longer chains. This effect will be confounded by both sampling bias (these chemicals are of more concern and are more heavily analyzed in the population) and environmental load (these are the most heavily manufactured and used class of PFAS, at least in the past).

**Figure 4:**
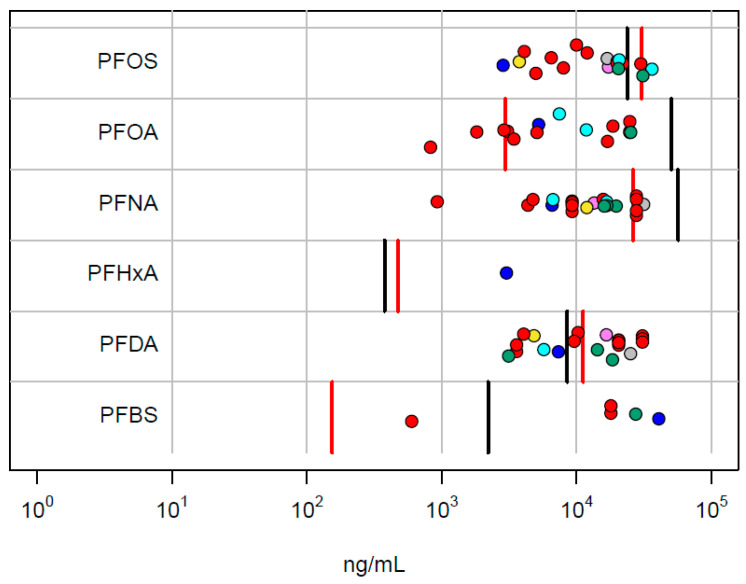
Comparison of in vitro POD_set_ values with internal concentrations corresponding to the lowest in vivo LEL values in the NTP study. Points correspond to the in vitro technologies (ACEA: violet; ATG: blue; BSK: red; HTPP: green; HTTr: cyan; Zebrafish: yellow; DNT: black; Thyroid: gray). The vertical lines are the POD concentrations for male (red) and female (black) rats.

As an independent validation of the relevance of the in vitro PODs, we compare these values with the internal concentration at the lowest in vivo LEL in the NTP rat study. The rat internal concentrations are from Table 1. These data are summarized in Figure 4. For 5 out of 6 chemicals, the in vitro POD_set_ overlaps the range of the male and female lowest LEL concentrations. With the exception of PFHxA, the lowest in vitro POD is below the in vivo LEL.

## 4. Discussion

We have demonstrated an approach to prioritizing chemicals for risk assessment based on the bioactive concentration-to-blood concentration ratio (BCBCR) between measured blood concentrations and effect concentration values from in vitro assays. An important caveat to this approach is that it relies on relatively stable blood concentrations, so it is most appropriate for chemicals that are long-lived in human tissues, which is the case for many PFAS compounds.

**Figure 5:**
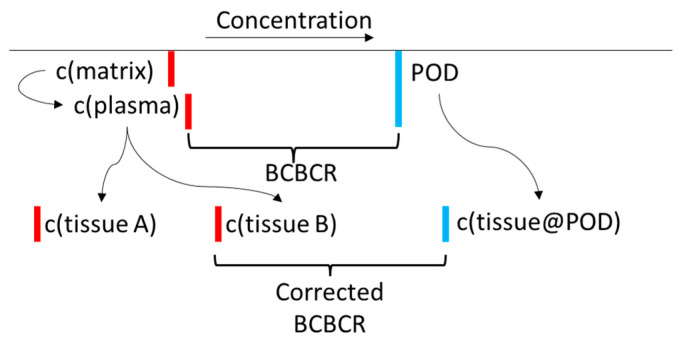
An illustration of the toxicokinetic corrections described in the text. The final BCBCR is c(aqueous@AC50)/max(c(tissue)). The lower the BCBCR value is, the more likely it is that the chemical will cause adverse effects in the measured population. Red lines indicate chemical concentrations in the body, and blue lines indicate bioactivating concentrations. Note that the tissue doses could increase or decrease after TK corrections.

Ideally, in this type of analysis, one would correct the blood and in vitro concentrations for TK factors. Biomonitoring studies measure concentrations in plasma, serum, or whole blood matrices, but the toxic effects from chemical exposure may occur in other tissues. Two kinds of corrections could be carried out. In the first, one would run a TK model to estimate tissue concentrations using the measured plasma concentrations but incorporating tissue-to-plasma partitioning. As evaluation of in utero exposure is important for some PFAS, additional modeling to include cross-placental transport may be warranted. When considering the relevant effect to compare with target tissue concentrations, one would then consider which biological target would be relevant for comparison to that specific tissue concentration in a BCBCR evaluation. Also, adjustments to the nominal applied concentrations that account for sequestration or migration into different compartments of the in vitro system (e.g., cells, media constituents, plastic, headspace) may be required to adjust the relevant concentrations at which bioactivity was observed [68,69]. Figure 5 shows the basic scheme required to carry out these corrections. One would then have to consider if, for a certain tissue endpoint, in vitro disposition could be used to estimate a target tissue concentration that corresponds to the appropriate in vivo tissue levels. All of these factors are subject to modeling and parameter uncertainty for any chemical, but the properties of PFAS increase the uncertainty. Regardless, we carried out these corrections using the *httk* R package [35] which applies generic TK models based on two experimental parameters (plasma protein binding and intrinsic clearance). The results (not shown) differ from the uncorrected results presented here in detail, but differences in overall trends (e.g., ranking of chemicals by minimum BCBCR) are not seen.

In a scenario of ideal data availability, an BCBCR < 1 suggests potential health risk because estimated tissue-level bioactivity coincides with reported exposure levels. However, there are multiple sources of uncertainty that need to be considered, and these could push the true BCBCR to lower values (higher potential risk) or higher values (lower potential risk). These will be considered in turn:

1. Toxicokinetics (TK): There are multiple uncertainties associated with toxicokinetics, some already described. For some PFAS, there are active transport mechanisms that could increase or, more typically, decrease excretion [5]. Further complicating in vitro-in vivo extrapolation. TK uncertainties could cause BCBCR values to increase or decrease.
2. In Vitro Disposition: As already mentioned, one would need to add a correction for chemical-specific in vitro distribution within the bioactivity testing well. These uncertainties could cause BCBCR values to increase or decrease.
3. Incomplete coverage of bioactivity assays/mechanisms. The current study uses a selected battery of in vitro assays that cover a relatively targeted set of biological processes. Adding more assays could potentially make the in vitro PODs decrease. This uncertainty would only cause the BCBCR to decrease.
4. Uncertainty in in vitro PODs for existing assays. All in vitro assays are subject to noise, and a variety of assay artifacts that can cause false positives or false negatives. As a rule of thumb, if a particular target was evaluated in multiple assays using different technologies, the POD could range by about an order of magnitude [70]. These uncertainties could cause BCBCR values to increase or decrease.
5. Not all PFAS have long half-lives. For chemicals with short half-lives, blood draws with timing unrelated to exposures will tend to underestimate peak concentrations in individuals or populations [71]. This uncertainty would only cause the BCBCR to decrease.
6. Bioactivity is not necessarily toxicity. The in vitro assays used here (and many others that one might use) measure perturbations in biology that might not lead to apical toxicity. There can be compensatory or adaptive mechanisms to prevent overt toxicity. Overt toxicity may require that the tissue concentration exceed a threshold level for extended periods of time or that the effect concentration be reached at a particular life stage. These uncertainties would only cause BCBCR values to increase.
7. Uncertainties in the blood measurements: The blood concentration measurements are themselves subject to uncertainty, although analytical techniques for PFAS have significantly improved with time. So these uncertainties are likely smaller than some of the others mentioned. Regardless, this uncertainty could cause BCBCR values to increase or decrease.
8. Different populations have different exposures and, therefore, different blood levels. Also, different sampled individuals with the same exposure can have different blood levels due to lifestage, genetic, and environmental factors. To estimate this uncertainty, consider the 50th percentile data for PFOA or PFOS in Figure 1. These data comprise mean and median values from many population samples, including individuals known to be exposed and individuals from the general population. Values span many orders of magnitude, with significant density of values over ~2 orders of magnitude. The higher values tend to be from exposed populations (e.g., workers in factories manufacturing PFAS, firefighters using PFAS foams, individuals consuming fish from PFAS-contaminated water, individuals drinking water from PFAS-contaminated wells), but there are outlier values from (supposedly) non-exposed populations. Regardless, as more populations are tested, minimum BCBCR values can only decrease.
9. Other PFAS may not have been identified. The PFAS that have been tested for in blood may be the original (manufactured) parent compound, or they could be degradates or human metabolites. The presence of one PFAS may indicate that others are also present, and these may not be detected either because they are short-lived (but not necessarily nontoxic) or not tested for. The original exposure could also be to a mixture of PFAS, including parents and degradates. This uncertainty would only cause BCBCR values to decrease for the measured PFAS, not necessarily for the initially manufactured and released compound.

Overall, there are enough uncertainty factors that would cause BCBCR estimates to decrease (increasing risk) that it would be prudent to set the level of concern at a higher BCBCR level than 1, and a factor of 100–1000 might be a reasonable approach to prioritizing further study of a chemical. This is consistent with screening-level assessment practices under TSCA (Toxic Substances Control Act). As with uncertainty factors used in traditional risk assessments, typical magnitudes of the factors above need to be estimated. There are several approaches to address these uncertainties, some of which our group is pursuing. As part of the project that produced the in vitro data used here, analytical methods for a large number of PFAS were developed, and we plan to test blood samples from both exposed and general populations against that larger set of analytes. Some of our collaborators have already begun using non-targeted analysis (NTA) to look for further, unknown PFAS analytes [72], and applying NTA to biomonitoring samples is part of our future plans. Next, it will be useful to test for further types of biological perturbations in these PFAS. One example that we are developing is a suite of immunotoxicity assays to address the reported immunotoxic effects of certain PFAS. A key piece of the TK analysis that we currently lack is understanding active transport of PFAS, both when it occurs and the magnitude of this effect on clearance and therefore tissue concentrations. In vitro evaluations of hepatic, intestinal, and renal transporter involvement across over 50 PFAS are underway now and will be used to refine PFAS IVIVE modeling in the near future.

We need to reiterate the point that the current approach is restricted to chemicals with long half-lives (months to years), as is the case with many PFAS. For these chemicals, the concentrations in blood will be relatively constant in time (hour-to-hour, day-to-day), which lends stability to the BCBCR value. Blood levels for chemicals with shorter half-lives will vary considerably depending on the duration between exposure and measurement.

In summary, the BCBCR method provides a practical approach to carrying out screening-level risk assessments on compounds like many PFAS that have long half-lives in human tissues. Because of the inherent uncertainties listed above, an initial use of this method could be for prioritization, helping to answer the question of which chemicals to focus on for more traditional risk assessments. An interesting contrast can be made between the BCBCR method and standard, animal-based methods. In both cases, accurate exposure estimates can be made using blood levels. In the animal-based approach, one would estimate an effect level (BMD, LOAEL) from experimental species and then carry out allometric and toxicokinetic corrections to estimate human blood levels at the effect level. The HQ [24] and the biomonitoring equivalent [26,27] methods described in the introduction follow such approaches. These methods also have uncertainties, the first being understanding potential differences in the effect of the chemical on the experimental animals vs. humans. The toxicokinetics can also be significantly different between experimental animals and humans. As an example, the half-life of PFOS is estimated to be 4.8 years in humans and 1-2 months in rodent species [73]. In summary, we believe that this approach can be useful for prioritizing PFAS compounds (and other chemistries with long half-lives) for further assessment.

## Figures and Tables

**Table 1:** NTP In Vivo Data. The effect levels are all given in mg/kg-day. The phenotype columns provide the LEL values in mg/kg-day.

Name	Sex	Liver Weight	Relative Liver Weight	Kidney Weight	Relative Kidney Weight	Decreased Hematocrit	Decreased Cholesterol	Decreased t3	Decreased Free t4	Decreased Total t4	Plasma Conc. at Lowest LEL (ng/mL)
PFBS	female	250	125	NA	62.6	NA	500	62.6	62.6	62.6	154.3
PFBS	male	125	62.6	500	500	62.6	62.6	62.6	62.6	62.6	2222
PFDA	female	0.156	0.156	0.312	0.625	1.25	1.25	NA	1.25	NA	11,207.8
PFDA	male	0.156	0.156	2.5	0.625	NA	0.156	0.312	0.312	0.312	8505
PFHxA	female	500	500	1000	1000	250	250	NA	NA	NA	475.4
PFHxA	male	500	250	NA	500	62.6	62.6	62.6	62.6	62.6	378.2
PFHxSK	female	3.12	3.12	NA	NA	NA	NA	NA	6.25	12.5	37,030
PFHxSK	male	1.25	1.25	NA	10	NA	1.25	0.625	0.625	0.625	66,760
PFNA	female	1.56	1.56	1.56	1.56	NA	NA	3.12	3.12	3.12	26,400
PFNA	male	0.625	0.625	2.5	1.25	NA	0.625	0.625	0.625	0.625	56,730
PFOA	female	25	25	50	100	6.25	50	NA	100	100	2960.1
PFOA	male	0.625	0.625	1.25	0.625	1.25	1.25	0.625	0.625	0.625	50,690.2
PFOS	female	0.312	0.312	NA	NA	NA	5	0.312	0.312	0.625	30,530
PFOS	male	0.312	0.312	NA	NA	NA	0.312	0.625	0.312	0.312	23,730

**Table 2:** Chemicals with biomonitoring, in vitro, and blood-to-plasma partitioning data.

DTXSID	CASRN	Name	Abbreviation
DTXSID20874028	914637-49-3	2H,2H,3H,3H-Perfluorooctanoic acid	5:3 PFOA
DTXSID6027426	1691-99-2	2-Perfluorooctylsulfonyl-N-ethylaminoethyl alcohol	N-EtFOSE
DTXSID90382620	812-70-4	3-(Perfluoroheptyl)propanoic acid	7:3 FTCA
DTXSID00379268	356-02-5	3:3 Fluorotelomer carboxylic acid	3:3 FTCA
DTXSID30891564	757124-72-4	4:2 Fluorotelomer sulfonic acid	4:2 FTSA
DTXSID90558000	57678-01-0	6:2 Fluorotelomer phosphate monoester	6:2 monoPAP
DTXSID6067331	27619-97-2	6:2 Fluorotelomer sulfonic acid	6:2 FTSA
DTXSID00192353	39108-34-4	8:2 Fluorotelomer sulfonic acid	8:2 FTS
DTXSID8037708	3825-26-1	Ammonium perfluorooctanoate	PFOAA
DTXSID1032646	4151-50-2	N-Ethylperfluorooctanesulfonamide	NEtFOSA
DTXSID7027831	24448-09-7	N-Methyl-N-(2-hydroxyethyl)perfluorooctanesulfonamide	N-MeFOSE
DTXSID1067629	31506-32-8	N-Methylperfluorooctanesulfonamide	N-MeFOSA
DTXSID70880215	13252-13-6	Perfluoro-2-methyl-3-oxahexanoic acid	GenX
DTXSID5030030	375-73-5	Perfluorobutanesulfonic acid	PFBS
DTXSID4059916	375-22-4	Perfluorobutanoic acid	PFBA
DTXSID3031860	335-76-2	Perfluorodecanoic acid	PFDA
DTXSID8059920	375-92-8	Perfluoroheptanesulfonic acid	PFHpS
DTXSID1037303	375-85-9	Perfluoroheptanoic acid	PFHpA
DTXSID50469320	41997-13-1	Perfluorohexanesulfonamide	FHxSA
DTXSID7040150	355-46-4	Perfluorohexanesulfonic acid	PFHxS
DTXSID3031862	307-24-4	Perfluorohexanoic acid	PFHxA
DTXSID8031863	375-95-1	Perfluorononanoic acid	PFNA
DTXSID3038939	754-91-6	Perfluorooctanesulfonamide	PFOSA
DTXSID3031864	1763-23-1	Perfluorooctanesulfonic acid	PFOS
DTXSID8031865	335-67-1	Perfluorooctanoic acid	PFOA
DTXSID6062599	2706-90-3	Perfluoropentanoic acid	PFPeA
DTXSID8059970	422-64-0	Perfluoropropanoic acid	PFPrA
DTXSID3059921	376-06-7	Perfluorotetradecanoic acid	PFTeDA
DTXSID90868151	72629-94-8	Perfluorotridecanoic acid	PFTriDA
DTXSID8047553	2058-94-8	Perfluoroundecanoic acid	PFUnDA
DTXSID3037707	29420-49-3	Potassium perfluorobutanesulfonate	KPFBS

## Data Availability

All data for this article is available in the Supplemental Material.

## Supplementary Materials

There are multiple supplemental files. These are described in the file S0—PFAS Biomonitoring Supplemental Material.docx. All supplemental data are available at the following URL: https://doi.org/10.23645/epacomptox.24645711.
